# Delimiting the Origin of a B Chromosome by FISH Mapping, Chromosome Painting and DNA Sequence Analysis in *Astyanax paranae* (Teleostei, Characiformes)

**DOI:** 10.1371/journal.pone.0094896

**Published:** 2014-04-15

**Authors:** Duílio M. Z. de A. Silva, José Carlos Pansonato-Alves, Ricardo Utsunomia, Cristian Araya-Jaime, Francisco J. Ruiz-Ruano, Sandro Natal Daniel, Diogo Teruo Hashimoto, Cláudio Oliveira, Juan Pedro M. Camacho, Fábio Porto-Foresti, Fausto Foresti

**Affiliations:** 1 Departamento de Morfologia, Instituto de Biociências, Universidade Estadual Paulista, Distrito de Rubião Junior, Botucatu, São Paulo, Brazil; 2 CAUNESP, Universidade Estadual Paulista, Campus Jaboticabal, Jaboticabal, São Paulo, Brazil; 3 Departamento de Genética, Universidad de Granada, Granada, Spain; 4 Departamento de Ciências Biológicas, Faculdade de Ciências, Universidade Estadual Paulista, Campus de Bauru, Bauru, São Paulo, Brazil; Virginia Tech, United States of America

## Abstract

Supernumerary (B) chromosomes have been shown to contain a wide variety of repetitive sequences. For this reason, fluorescent in situ hybridisation (FISH) is a useful tool for ascertaining the origin of these genomic elements, especially when combined with painting from microdissected B chromosomes. In order to investigate the origin of B chromosomes in the fish species *Astyanax paranae*, these two approaches were used along with PCR amplification of specific DNA sequences obtained from the B chromosomes and its comparison with those residing in the A chromosomes. Remarkably, chromosome painting with the one-arm metacentric B chromosome probe showed hybridization signals on entire B chromosome, while FISH mapping revealed the presence of H1 histone and 18S rDNA genes symmetrically placed in both arms of the B chromosome. These results support the hypothesis that the B chromosome of *A. paranae* is an isochromosome. Additionally, the chromosome pairs Nos. 2 or 23 are considered the possible B chromosome ancestors since both contain syntenic H1 and 18S rRNA sequences. The analysis of DNA sequence fragments of the histone and rRNA genes obtained from the microdissected B chromosomes showed high similarity with those obtained from 0B individuals, which supports the intraspecific origin of B chromosomes in *A. paranae*. Finally, the population hereby analysed showed a female-biased B chromosome presence suggesting that B chromosomes in this species could influence sex determinism.

## Introduction

About 15% of eukaryotic species carry supernumerary (B) chromosomes, i.e. dispensable genomic elements present in only some individuals from a given population. In most cases, B chromosomes behave as parasitic elements prospering in natural populations due to their advantage in transmission (drive) not obeying Mendelian rules [1]–[6]. In some cases, B chromosomes appear to provide some advantage to the host genome and can thus be considered heterotic [7], [8]. Examples of advantageous B chromosomes were already reported in the *Nectria haematococca* with B chromosomes favouring this fungus pathogenicity [9] and in the chive *Allium schenoprassum*, in which the B chromosomes increase viability from seed to seedling [10].

Historically, B chromosomes were considered inert genomic elements [11], but recent research has shown the transcription of several types of genes residing in B chromosomes, such as rRNA genes [12], [13], repetitive DNA with similarity to mobile elements [14] and protein-coding genes in the Siberian roe deer *Capreolus pygargus* [15]. Interestingly, B chromosomes in a cichlid fish species have been shown to have a functional effect on female sex determination, with B chromosomes being restricted to females [16]. This effect is similar to the case of the paternal sex ratio (PSR) chromosome in wasps, in which a B chromosome has the opposite effect, i.e., transforming female determining zygotes into males and B chromosomes thus being restricted to males [6], [17].

DNA composition of B chromosomes is unknown in most cases, even though this is an interesting topic in B chromosome research. In general, B chromosomes are rich in several classes of repetitive DNA, including 5S and 45S ribosomal DNA (rDNA), satellite DNA, histone genes, small nuclear DNA, mobile elements, and organellar sequences [11], [18]–[25]. Chromosome painting has been used in several species to ascertain whether B chromosomes share DNA sequences with the A chromosomes from the same or other closely related species [20], [26]–[32]. In addition, fluorescent in situ hybridisation (FISH) mapping of repetitive DNA has also provided valuable information in some cases [21], [31], [33]–[36]. Recently, next generation sequencing (NGS) technology has been applied to B chromosome research in three species: the fungus *Mycosphaerella graminicola* [37], the fruit fly *Drosophila albomicans* [38] and rye *Secale cereale* [39]. This approach has shown that the B chromosome content is a complex mixture of different types of DNA sequences, including the already known types of repetitive DNAs (see above), many insertions of organellar DNA and many pseudogenized single-copy genes, which may be transcribed [39], [40].

Although B chromosomes have been reported in a variety of fish groups [41], few studies using a multiple molecular and cytogenetic approach have been carried out. Among fishes, the genus *Astyanax* represents an interesting model to study B chromosomes, with occurrence records in ten species [41]–[47]. However, significant data about origin and evolution of those chromosomes are restricted to the species *A. scabripinnis* [31], [33].

Aiming to enhance the knowledge about composition, origin, evolution and possible function of B chromosomes in *A. paranae*, we performed chromosome painting with B-DNA microdissected probes and FISH mapping with different repetitive DNA probes (5S and 18S rDNA; H1, H3, and H4 histone genes; and *Rex1* and *Rex3* transposable elements). Moreover, we analysed the nucleotide sequences of ribosomal and histone multigene families obtained from microdissected B chromosomes and from genomic DNA of B-lacking individuals of *A. paranae* and three other *Astyanax* species.

## Materials and Methods

### Ethics Statement

Samples were collected in accordance with Brazilian environmental protection legislation (collection permission MMA/IBAMA/SISBIO - number 3245), and the procedures for sampling, maintenance and analysis of the samples were performed in compliance with the Brazilian College of Animal Experimentation (COBEA) and was approved (protocol 405) by the BIOSCIENCE INSTITUTE/UNESP ETHICS COMMITTEE ON USE OF ANIMALS (CEUA).

### Origin of samples and karyotypic analysis

We randomly collected 50 individuals (18 males and 32 females) of the fish species *Astyanax paranae* Eigenmann, 1914 [48] and some individuals without B chromosomes of *A. bockmanni*, *A. fasciatus* and *A. altiparanae*, in the Capivara River belonging to the Tietê River basin (22°53′57′′S/48°23′11′′W), close to Botucatu (São Paulo, Brazil). The specimens were identified and deposited in the fish collection of the Laboratório de Biologia e Genética de Peixes de Botucatu, São Paulo, Brazil, under the numbers LBP13340 (*A. paranae*), LBP13342 (*A. bockmanni*), LBP13344 (*A. fasciatus*) and LBP13346 (*A. altiparanae*). To perform cytogenetic analyses, the specimens were anesthetised and dissected, and the mitotic chromosomes were obtained from kidney tissue and gills using the technique described in Foresti et al. [49]. C-banding was performed following the protocol described by Sumner [50], and the nucleolar organiser regions (NORs) were identified according to Howell and Black [51]. Chromosome morphology was determined according to the criterion established by Levan et al. [52], and thus, the morphology was classified as metacentric (*m*), submetacentric (*sm*), subtelocentric (*st*), and acrocentric (*a*) and was arranged in the karyotype in a decreasing size order.

### Chromosome microdissection

Microdissection was performed in an Eppendorf TransferMan NK2 micromanipulator coupled to a Zeiss Axiovert 100 microscope. We microdissected ten copies of a complete metacentric B chromosome (Bm) for DNA sequencing (µB-DNA). Moreover, two different B-derived DNA probes were generated through microdissection for FISH experiments: 1) one arm of one copies of the Bm chromosome (µBm-probe) (in order to test its isochromosome nature) and 2) ten copies of a complete submetacentric B chromosome (Bsm) (µBsm-probe). The microdissected DNAs were placed in 9 µl of DNase-free ultrapure water and then amplified using the GenomePlex Single Cell Whole Genome Amplification Kit (wga4-Sigma) [53]. After the initial amplification, we generated DNA probes (µBm-probe and µBsm-probe) labelled with digoxigenin-11-dUTP (Roche Applied Science) using the GenomePlex Whole Genome Amplification Reamplification Kit (wga3-Sigma) following the manufacturer's protocol.

### DNA amplification, cloning and sequencing

For gDNA extraction, we used the Wizard Genomic DNA Purification Kit (Promega) following manufacturer's instructions. For taxonomic confirmation of all materials collected, we performed DNA barcoding by amplifying a partial sequence of the mitochondrial gene Cytochrome c oxidase subunit 1 (COI), with the primers: FishF1 5′-TCAACCAACCACAAAGACATTGGCAC-3′ and FishR1 5′-TAGACTTCTGGGTGGCCAAAGAATCA-3′ [54].

Partial DNA sequences for 5S and 18S rDNA, ITS, H1, H3, and H4 histone genes, and *Rex1* and *Rex3* transposable elements (TEs) were obtained by PCR (Polymerase Chain Reaction) from *A. paranae* genomic DNA (gDNA) lacking B chromosomes using the primers described in Table S1. The reactions were performed in 1X PCR buffer, 1.5 mM of MgCl_2_, 200 µM of each dNTP (dATP, dCTP, dGTP, dTTP), 0.5 U of *Taq* polymerase (Invitrogen), 0.1 µM of each primer and 50 ng of gDNA. The basic cycle to amplify these regions consisted of denaturation at 95°C for 5 min, followed by 30 cycles at 95°C for 1 min, 45 s at T annealing °C (Table S1), 1 min at 72°C and a final extension of 10 min at 72°C.

The 18S rDNA, the ITS regions, and the H1 and H3 histone genes were amplified on the *A. paranae* µB-DNA, and we also amplified them on 0B-gDNA from *A. paranae*, *A. bockmanni, A. fasciatus* and *A. altiparanae*. To isolate a representative diversity of copies of these sequences present in the PCR reaction, we cloned the PCR-obtained bands for these genes by linking them to a TOPO TA cloning vector and cloning them in One Shot TOP10 Competent Cells. A number of clones were chosen for DNA sequencing. We then isolated the plasmid DNA with the Perfectprep Plasmid Mini kit (Eppendorff). For all DNA sequences, the PCR products were purified using the ExoSAP-IT kit (USB Corporation) and were sequenced with the Big Dye TM Terminator v 3.1 Cycle Sequencing Ready Reaction Kit (Applied Biosystems), following manufacturer's instructions. We sequenced each plasmid in both directions using the M13F (5′-GTAAAACGACGGCCAG-3′) and M13R (5′-CAGGAAACAGCTATGAC-3′) primers.

### Fluorescent *in situ* hybridisation (FISH)

For FISH experiments, the chromosomes were treated according to the procedures described by Pinkel et al. [55], using high stringency conditions. The probes were labelled by PCR with biotin-16-dUTP (Roche Applied Science) and the signal was detected with avidin-FITC (Roche Applied Science), or else they were labelled with digoxigenin-11-dUTP (Roche Applied Science) and the signal was detected with anti-digoxigenin-rhodamine (Roche Applied Science). The images were captured with a digital camera (Olympus DP70) attached to an Olympus BX61 epifluorescence photomicroscope. Image treatment, including karyotype mounting and optimisation of brightness and contrast, was performed using the Adobe Photoshop CS4 program.

### Sequence analysis

All DNA sequences were initially analysed with Geneious version Pro 4.8.5 created by Biomatters (http://www.geneious.com/) and sequence alignments were performed with Clustal W [56] implemented in Geneious software. DNA diversity analyses were performed with the DnaSP software [57]. Calculation of dN and dS, and selection tests for protein coding DNA sequences were performed with the MEGA software [58] using the Nei-Gojobori method [59]. We built maximum likelihood trees for the 18S, H1, H3 and ITS regions using the PhyML software [60] considering a GTR + G model and performing a bootstrap analysis with 1000 replicates. A p-distance matrix among groups was performed for the concatenated ITS sequences using pairwise deletion for missing data. We inferred a species tree with all obtained sequences from the gDNA of the *Astyanax* species and the B chromosome of *A. paranae* for all the regions with two or more clones by group, i.e, H1, H3 and the ITS, using the BEAST software [61]. We launched three MCMC during 10^8^ generations, sampling a tree each 1000 generations. We assessed the convergence of the three runs with Tracer and combined the resulting trees with LogCombiner after applying a 10% burnin.

In order to confirm their identity, the sequences were used as queries for BLASTn [62] searches against NCBI’s nr database (http://www.ncbi.nlm.nih.gov/blast) and then were deposited in the GenBank with the following accession numbers: KJ129765 (*Rex1*), KJ129638-KJ129645 (COI sequence), KJ129646-KJ129668 (H1 histone gene), KJ129669-KJ129692 (H3 histone gene), KJ129693-KJ129728 (ITS1 sequence), KJ129729-KJ129764 (ITS2 sequence) and KJ129623-KJ129637 (18S rDNA). For *A. bockmanni* species identification, we used COI sequences deposited in GenBank under the accession numbers KC153991-KC153994. The COI sequences were submitted to the identification engine tool from BOLDSYSTEMS (http://www.barcodinglife.com/index.php/IDS_OpenIdEngine). All sequences contaminated with ambient bacterial DNA were carefully screened and discarded. Statistical comparisons by chi-square and Student t tests were performed in a spreadsheet program.

## Results

**Figure 1:**
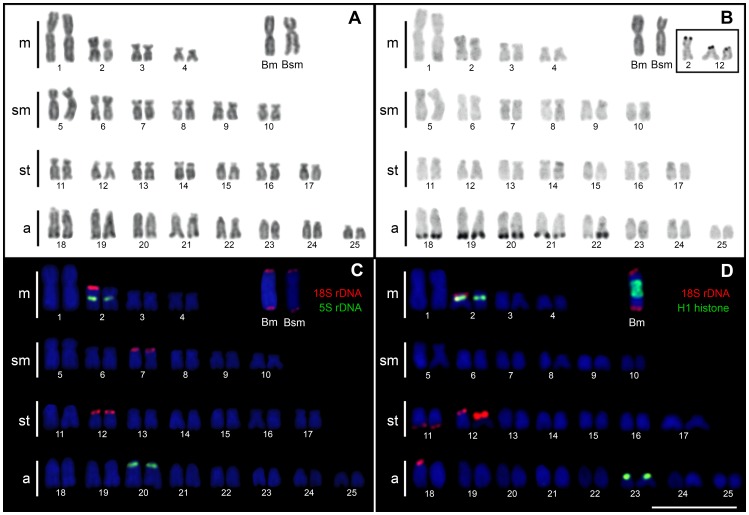
Karyotypes of *Astyanax paranae* from the Capivara River after conventional staining (A), C-banding (B), and double FISH with 5S and 18S rDNA probes (C) and 18S rDNA and H1 histone (D). Ag-NORs are represented in the box. Bar = 10 µm.

The molecular identification engine tool showed that the COI region amplified from the analysed samples correspond to *A. paranae*, *A. bockmanni*, *A. fasciatus* and *A. altiparanae* with 100% of similarity in all cases. The analysed samples of *A. paranae* showed 2n = 50 chromosomes (8m+12sm+14st+16a, NF = 84) (Figure 1a). Moreover, B chromosomes were observed in 100% of the cells in 12 out of 32 females, ten of which carried a metacentric macro B (Bm) and two carried a submetacentric macro B (Bsm) (Figure 1a). Only one out of the 18 males analysed carried the Bm chromosome. No individual carried more than one B chromosome per cell. A contingency chi-square test showed that B frequency was nearly significantly higher in females (χ^2^ = 3.67, df = 1, P = 0.0555).

### Chromosome analysis

C-banding technique showed two types of heterochromatin: faint C-positive blocks were present in some A chromosomes and in the B chromosomes, while strong C-positive blocks were present in the terminal region of the long arms of the acrocentric pairs Nos. 18–22 (Figure 1b). Ag-NORs showed intra-population variation, with pair No. 12 showing the principal NOR activity, and pair No. 2 sometimes showing Ag-NOR positive signals (Figure 1b). No Ag-NORs were observed in the B chromosomes.

**Figure 2:**
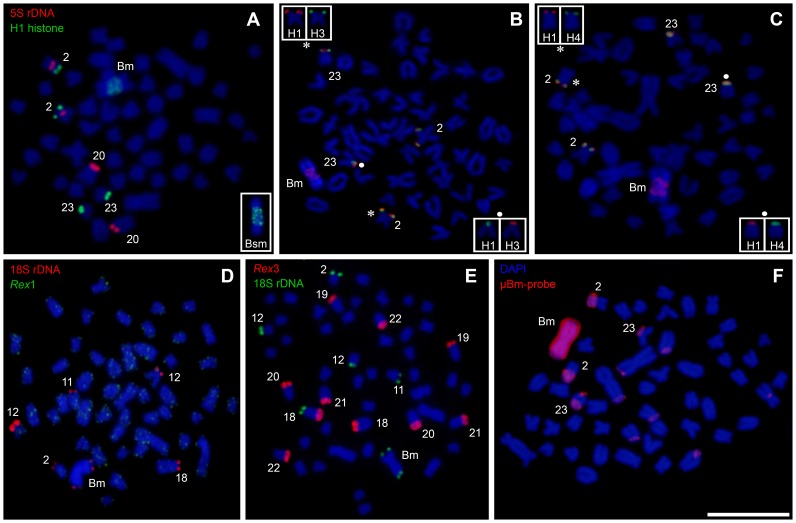
Metaphases of *Astyanax paranae* from the Capivara River after double-FISH with 5S rDNA and H1 histone (A), H1 and H3 histone (B), H1 and H4 histone (C), *Rex1* and 18S rDNA (D), *Rex3* and 18S rDNA (E) and after chromosome painting with µBm-probe (F). Bar = 10 µm.

**Figure 3:**
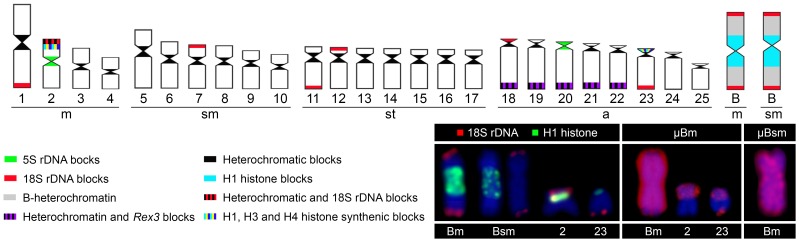
Ideogram of *Astyanax paranae* from the Capivara River showing the main cytogenetic markers localisation used in this paper. In the box, a summary of the FISH markers and chromosome painting associated with B chromosome in chromosomes 2, 23, Bm and Bsm.

Consistent with Ag-NOR patterns, chromosome pair No. 12 carried an 18S rDNA cluster in the short arm. Additionally, both types of B chromosomes carried a small rDNA cluster in the terminal region of both chromosome arms (Figures 1c,d, 2d,e). We also observed the variable presence of rDNA clusters in six other A chromosomes, namely pairs Nos. 1, 2, 7, 11, 18 and 23 (Figures 1c,d, 3); however, likewise Ag-NOR, only the rDNA in chromosome 2 was observed as an active NOR (Figure 1b, inset).

The 5S rDNA was located in the pericentromeric region of the pairs Nos. 2 and 20 (Figures 1c, 2a). H1, H3 and H4 histone sites were found in synteny on the short arms of chromosomes 2 and 23 (Figures 1d, 2a, 2b, 2c). Also, H1 histone genes were detected in Bm chromosome pericentromeric region distributed symmetrically in both arms. There was an unequal distribution of the H1 histone genes in Bsm chromosome, with most of them located in the long arm (Figure 2a). The *Rex1* transposable element was scattered throughout all A chromosomes (Figure 2d), but *Rex3* was compartmentalised in the heterochromatic regions of the acrocentric chromosomes no. 18–22 (Figure 2e).

The chromosome painting technique yielded similar patterns with the two probes assayed (µBm-probe and µBsm-probe), with the Bm and Bsm chromosomes being completely marked along with small signals in some A chromosomes (Figure 2f). Remarkably, some of these signals were coincident with ribosomal and histone sites, mainly in the short arms of pairs Nos. 2 and 23 (Figure 2f). All of the information obtained by chromosome painting and FISH mapping is summarised in the ideogram in Figure 3.

### DNA sequence analysis

We tried to amplify several types of repetitive DNA (18S, ITS1 and ITS2 regions of the 45S rDNA, 5S rDNA and H1, H3 and H4 histone genes) from 0B genomic DNA (0B-gDNA) and from microdissected B-DNA (µB-DNA). All these repetitive DNAs were amplified from 0B-gDNA, but only 18S, ITS H1 and H3 were amplified from microdissected DNA from B chromosome. We got the full sequence of all cloned regions. However, due to the length of ITS region (1800 bp), about 250 bp of the ITS2 region could not be included in the analysis.

A comparison of nucleotide diversity for the DNA sequences found in the microdissected B chromosomes with those obtained from 0B individuals showed significant differences for 18S rDNA, the ITS1 region and the H3 histone gene, but not for the ITS2 region or the H1 histone gene (Table 1). Whereas ITS1 and H3 showed lower nucleotide diversity in the B chromosome copies, the 18S rDNA region showed higher diversity in the B chromosome copies.

**Table 1:** Nucleotide diversity (π) for the DNA sequences analysed, and Student (t) tests comparing A chromosome (0B-gDNA) and microdissected B chromosome (µB) sequences.

Gene	Sample	n	Sites	S	Hap	π	SE	t	df	P
18S	0B-gDNA	8	1338	8	6	0.0019	0.0002			
	µB	5	1338	21	5	0.0072	0.0007	−7.48	11	0.00001
ITS1	0B-gDNA	5	428	20	4	0.0250	0.0033			
	µB	10	422	10	8	0.0051	0.0003	5.98	13	0.00005
ITS2	0B-gDNA	5	384	3	3	0.0032	0.0006			
	µB	10	384	6	5	0.0031	0.0004	0.04	13	0.97173
H1	0B-gDNA	8	451	22	7	0.0230	0.0020			
	µB	8	451	29	6	0.0172	0.0027	1.71	14	0.10860
H3	0B-gDNA	9	355	25	5	0.0174	0.0032			
	µB	7	355	8	5	0.0093	0.0008	2.43	14	0.02887

Where n =  number of sequences, S =  number of segregating sites, Hap =  number of haplotypes, SE =  standard error, df =  degrees of freedom, P =  probability.

Considering the H1 and H3 histone genes, the amplified regions included codons 14–163 for H1 and codons 4–121 for H3. Six out of the 150 aminoacid positions analysed in H1 showed variation, but no variation was found for H3. Additionally, no stop codons were observed for both genes. We calculated the number of synonymous and non-synonymous substitutions per synonymous (dS) and non-synonymous (dN) site, respectively, for each group of sequences. As shown in Table 2, no significant differences were observed between the DNA sequences coming from the A chromosomes (0B-gDNA) or the B chromosomes (µB). Both synonymous and non-synonymous substitutions were found for the H1 gene in both types of DNA sequences (0B-gDNA and µB), with dN/dS ratios being much lower than 1, but being three times higher for the DNA sequences coming from the B chromosome (Table 2), suggesting the possibility that purifying selection is relaxed for the H1 genes located in the B chromosomes. In fact, a comparison of the *A. paranae* H1 haplotypes with those obtained in the *Astyanax* species, by the Nei–Gojobori neutrality test, provided significant evidence for purifying selection (Table S2).

**Table 2:** Number of synonymous and non-synonymous substitutions per synonymous (dS) and non-synonymous (dN) site, respectively, observed in the DNA sequences of the H1 and H3 histone genes obtained from 0B genomic DNA (0B-gDNA) and B microdissected DNA (µB).

Gene	Sample	n	dN	SE	t	df	P	dS	SE	t	df	P	dN/dS
H1	0B-gDNA	8	0.004	0.002				0.077	0.016				0.052
	µB	8	0.007	0.002	−1.06	14	0.307	0.045	0.01	1.70	14	0.112	0.156
H3	0B-gDNA	9	0	0				0.068	0.011				0
	µB	7	0	0				0.036	0.012	1.97	14	0.069	0

**Figure 4:**
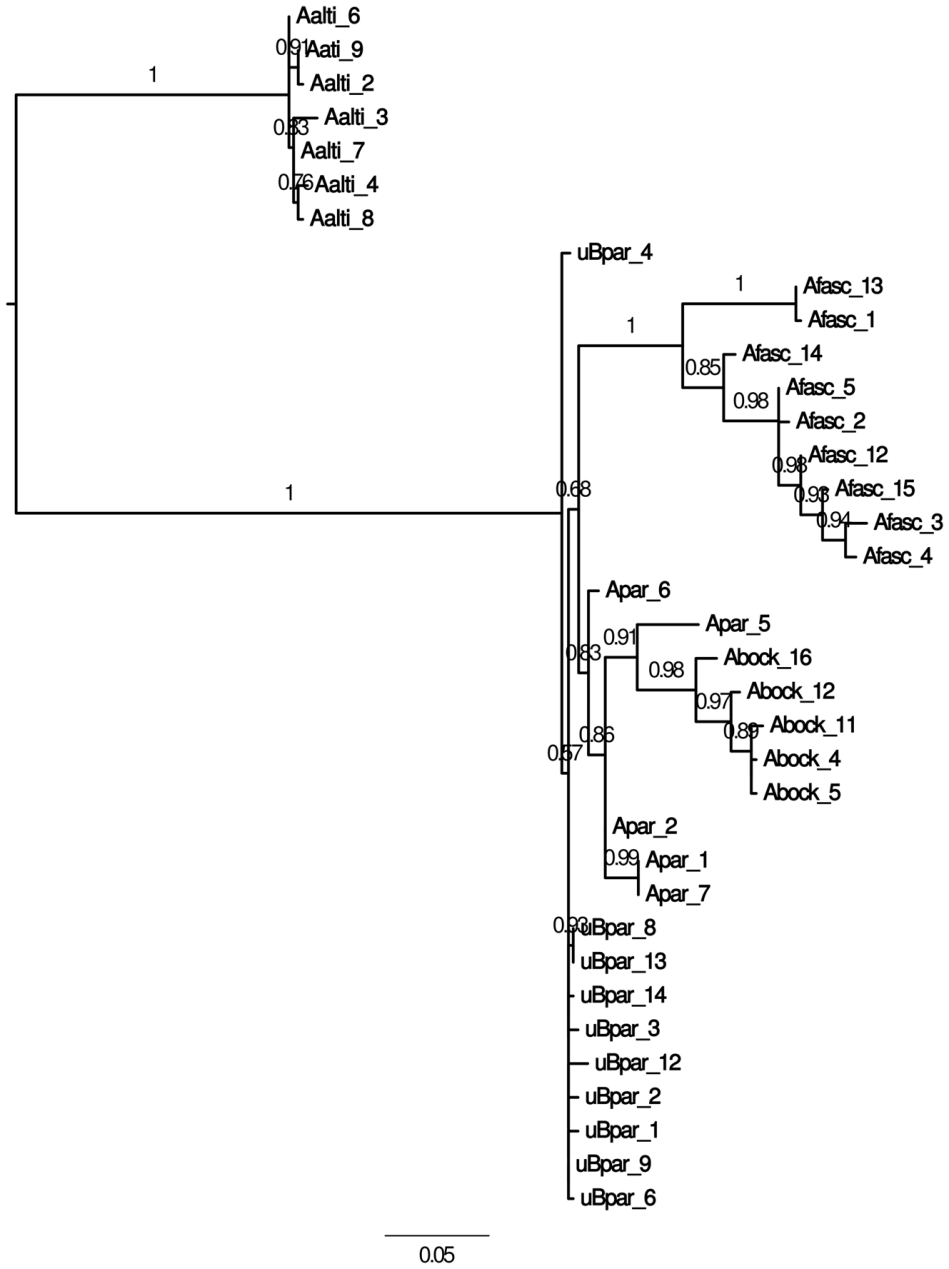
Maximum likelihood tree built with the concatenated ITS rDNA sequences. The sequences were obtained from the microdissected B chromosomes, gDNA from 0B *A. paranae* individuals, and gDNA from *A. bockmanni, A. fasciatus* and *A. altiparanae* (Aalti) specimens. Note the high similarity of the B chromosome sequences (uBpar) and the sequences obtained from 0B-gDNA in *A. paranae* (Apar) and *A. bockmanni* (Abock), and the lower similarity to those from *A. fasciatus* (Afasc).

**Figure 5:**
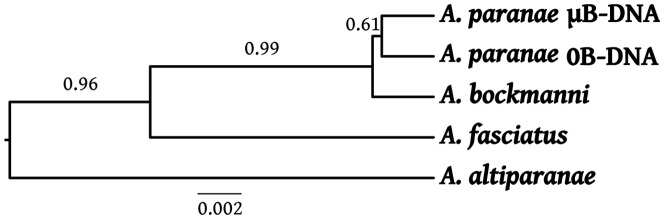
Species tree built with the histone genes and ITS regions by BEAST. Note that the DNA sequences of *A. paranae* obtained from the microdissected B chromosomes (µB-DNA) are most similar to those in the host genome (0B-gDNA).

Maximum-likelihood trees built with the sequences obtained from the microdissected B chromosomes, the 0B-gDNA from *A. paranae*, and gDNA from *A. bockmanni*, *A. fasciatus* and *A. altiparanae*, were not resolutive for 18S rDNA and the two histone genes due to the scarce variation observed (Figures S1–S3). Thus, concatenated ITS regions were used and suggested higher similarity between the sequences obtained from the B chromosomes and those from 0B-gDNA in *A. paranae* and *A. bockmanni*, and lower similarity with those from *A. fasciatus* and *A. altiparanae* (Figure 4). These conclusions are supported by the average p-distances shown in the Table S3. Additionally, a species tree performed by the BEAST method for the histone genes and the ITS regions indicated that the DNA sequences contained in the B chromosome of *A. paranae* are most similar to those in the A chromosomes of this same species, thus pointing to an intraspecific origin for the B chromosome (Figure 5).

## Discussion

B chromosomes in the genus *Astyanax* were first reported in *A. scabripinnis* as a large metacentric B chromosome similar to the largest A chromosome pair [63], [64]. Also, several B chromosomes have been found in different species within this genus, presenting variation in morphology, size and DNA composition [41], [42], [47]. In *A. paranae*, large B chromosomes have also been reported from the Tietê River basin [64], [65], but no compositional information had been reported yet.

Our results from chromosome painting with the DNA probes obtained from the Bm and Bsm chromosomes suggest that A and B chromosomes share similar DNA sequences. This finding suggests an intraspecific origin for this B chromosome (see 11) and points towards two A chromosomes (Nos. 2 and 23) as possible candidates to have been the B ancestor. However, this detected similarity may be attributed to the presence of large 18S rDNA and H1 histone clusters in these chromosomes. Although the anonymity of DNA probes used in the chromosome painting technique does not give great consistency to this conclusion, the combination of this technique with FISH mapping of repetitive DNAs, and the comparative analysis of such sequences from the microdissected B chromosomes, with those in the A chromosomes of *A. paranae* and other closely related species, has given strong support to the intraspecific origin for this B chromosome.

In fish, only satellite DNA, 45S rDNA and transposable elements had previously been found in the B chromosomes of *Astyanax scabripinnis* [33], *Prochilodus lineatus* [66], *Haplochromis obliquidens* [36], *Astatotilapia latifasciata* [67], *Alburnus alburnus* [68], while active nucleolar sites were observed in the B chromosomes of *Moenkausia sanctaefilomenae* [69]. Here, we show the first evidence of histone protein genes allocated in a fish B chromosome, a fact that had previously been reported only in the grasshoppers *Locusta migratoria* [21] and *Rhammatocerus brasiliensis* [22]. Also, both B chromosome types (Bm and Bsm) found in *A. paranae* carried 18S rDNA in the terminal region of both chromosome arms. The symmetric distribution of 18S rDNA and H1 histone genes in both arms suggests that the B chromosome is an isochromosome, as previously shown for a very similar macro B chromosome in *A. scabripinnis* [33]. Indeed, chromosome painting with the µBm-probe (which was obtained from a single B arm) painted the whole Bm and Bsm chromosomes, thus suggesting the homology between both B arms and the homology between both B types. In addition, the differences between the relative numbers of H1 histone genes in both arms of Bsm related to the Bm chromosome suggest that the origin of the former Bsm was via a pericentric inversion with asymmetrical breakpoints in the H1 regions of both arms. Remarkably, similar Bm and derived Bsm chromosomes have been reported in *A. scabripinnis* [31], [33], [42], [70]–[72].

The B chromosome origin through isochromosome in *A. paranae* implies that its two arms are homologous. Therefore, any explanation of B origin, in this case, needs to be limited to one arm only, as the final step of B origin is the centromeric misdivision of an acrocentric proto-B chromosome yielding a metacentric iso-B. In fact, the painting probe we generated from microdissection of a single chromosome arm of the metacentric B chromosome actually painted the two arms, thus demonstrating its isochromosome nature. We did not find useful information for 5S rDNA or *Rex* retrotransposons among the mapped repetitive DNA probes but our results with the 18S rDNA and histone genes clearly pointed to two A chromosomes as the most likely A chromosome ancestors of B chromosomes in this species. These were chromosomes Nos. 2 and 23 based on the remarkable fact that they are the only A chromosomes carrying the two repetitive DNAs (detected by FISH) found in the B chromosomes, i.e., 18S rDNA and H1 histone genes. These genes occupy distant locations in the B chromosomes: H1 being near the centromeric region (i.e., proximal) but 18S rDNA being close to the long arm telomere (i.e., distal). The comparison with these repetitive DNAs locations in chromosomes Nos. 2 and 23 indicates that the origin of the B chromosomes was not simple.

If the ancestor was chromosome 2, the B chromosomes could have derived from the short arm region containing the H1 and 18S rDNA, however, some additional DNA should have been placed in between these two sequences and the centromeric 5S rDNA should have been lost. Alternatively, the B chromosome could have derived from the long arm of chromosome 23, provided that it contains H1 histone genes in its proximal region; which would explain the presence of distal 18S rDNA and proximal H1 histone genes in the B chromosome, and the presence of DNA of unknown nature (the so-called B heterochromatin) between the two repetitive DNAs. This would suggest chromosome 23 as the most parsimonious hypothesis on the origin of B chromosomes. The absence of visible H3 and H4 histone sites in the B chromosomes, despite their syntenic condition with H1 in chromosomes 2 and 23 could be attributed to the occurrence of orphon genes (solitary genetic elements derived from tandem multigene families) that spread from this element [73]. Remarkably, a very similar A chromosome (no. 24) was suggested to be large metacentric B ancestor chromosome in *A. scabripinnis* [31], [33], [70], which suggests the possibility of a common origin for B chromosomes in these two species [74].

The higher dN/dS ratio of µB-DNA sequences compared with the A-chromosomes sequences (Table 2) indicates the possibility that purifying selection is relaxed for the H1 genes located in the B chromosomes, as expected if they were genetically inactive and could explain their differential amplification in the B chromosome. In fact, the lack of selective pressure on B chromosomes of rye allows the accumulation of *Revolver* repetitive DNA on these elements [75]. Conversely, the number of H3 copies in the B chromosome is probably lower, due to the absence of visible FISH signals. This could explain the lower nucleotide diversity observed in this gene of B chromosome sequences compared with those in the A chromosomes (Table 1).

The majority of the H1 and H3 histone sequences isolated from *A. paranae* B chromosomes showed the same putative amino acid sequence as those obtained from 0B genomic DNA, thus suggesting that they are potentially active. Teruel et al. [21] reported the presence of H3 and H4 histone sequences with the same putative amino acid sequences in the *L. migratoria* B chromosomes as those obtained from 0B genomic DNA indicating that they are potentially active. Ruiz-Estévez et al. [76] detected B-derived rRNA sequences in grasshopper *Eyprepocnemis plorans* confirming that B sequences are potentially active and can contribute to the host genome functions. Pseudogenic sequences of B chromosomes in rye are expressed and have potential to regulate the expression of A-located genes [40]. These examples show that B chromosomes are not always inert genomic elements, highlighting the necessity of future investigations about the potential activity of genes in *A. paranae* B chromosome.

The nucleotide diversity observed in the rDNA obtained from the B chromosome, compared with that shown by the same sequences in the A chromosomes unveiled some interesting facts. First, the 18S rDNA showed higher diversity in B chromosome than in A chromosomes, which would be consistent with the inactivity of B-rDNA. In fact, the Ag-NOR technique suggests that it is always inactive. On this basis, it should be expected that nucleotide diversity were also higher in B chromosome at ITS regions. However, it was about similar in the ITS2 and significantly lower in the ITS1 region (see Table 1). A possible explanation would be that the distal location of rDNA in both B chromosome arms, and the fact that iso-B-chromosomes frequently show distal chiasmata between the two homologous arms (which in the *A. paranae* B chromosome would involve the rDNA), would lead to the homogenization of the rDNA in both B chromosome arm ends through crossover, assuming that the B chromosome of *A. paranae* has the same behaviour on the meiosis as the B chromosome of *A. scabripinnis* [33]. However, in this work we did not analyse the meiosis behaviour of B chromosome due to the scarcity of male samples bearing B chromosomes. Moreover, this technique is a laborious task to apply in Teleostei females, since the pachytene phase occur in the early stages of the development [77]. Another possible explanation to the ITS nucleotide diversity in the B chromosome is based on the remarkable spreading of 18S rDNA in different chromosomes (Figures 1c, d, 2d, e). Thus, considering that interchromosomal homogenization rates are lower than intrachromosomal events [78], [79], the B-sequences (isolated from a single chromosome) might present a lower diversity when compared with 0B-gDNA (with several 18S rDNA A-chromosomes sites). Although these possibilities are very interesting, we realize that the limited number of DNA sequences analysed could also be the responsible for former contradictions; therefore further research with larger samples is needed.

The species tree built with the sequences of H1, H3 and ITS regions (Figure 5) was more informative than the gene trees (Figures 4, S1–S3) because it clearly separated the species *A. bockmanni* and *A. paranae* from the two other species (*A. altiparanae* and *A. fasciatus*), with posterior branch probability being higher than 0.95. In addition, this tree shows that *A. paranae* µB-DNA is closer to *A. paranae* 0B-gDNA indicating an intraspecific origin for this B chromosome. However, the posterior probability for the branch separating the 0B and µB sequences in *A. paranae* from those in *A. bockmanni* was only 0.61, thus casting some doubts about the topology obtained. This is probably due to the fact that *A. paranae* and *A. bockmanni* belong to the *A. scabripinnis* species complex which includes several closely related species sharing a very recent common ancestor, and this latter could already harbour the B chromosomes which could have be inherited by the descent species [74].

B chromosomes that are restricted to one sex have already been described in *Astyanax scabripinnis*, such as a micro B chromosome restricted to males [80] or a macro B restricted to females [81]. These cases were interpreted as the result of possible chromosome elimination from the somatic tissues analysed [80]. Of course, this possibility cannot be excluded without analysing the germ line in all of these cases. However, there is another interesting explanation focused on the possible effects of gene content in B chromosomes. The *A. paranae* sample collected in the Capivara River, analysed here showed a significant female biased sex ratio distortion because 32 individuals were female but only 18 were male (χ^2^ = 3.92, df = 1, P = 0.048). A previous sample of this same species by Maistro et al. [64] in the Araquá River (another tributary of the Tietê River) failed to find B chromosomes in males. Although Porto-Foresti et al. [65] found a female biased sex ratio showing B chromosomes present in females 57.1% and males 8.7% at the same collection site. A similar scenario has been observed for the large B chromosomes in *A. scabripinnis* [70]. The female-biased B chromosome presence in *A. paranae* shown here suggests the possibility that B chromosomes in this species influence sex determining. Remarkably, Yoshida et al. [16] have recently provided evidence that B chromosomes in a cichlid fish species exert functional effects on sex determination. Therefore, it would be interesting to investigate the presence of possible genes related with sex determinism in the *A. paranae* B chromosomes, and their possible activity during the embryo stages where sex determination is occurring.

## Supporting Information

Figure S1 Maximum likelihood tree built with the 18S rDNA sequences. The sequences were obtained from the microdissected B chromosomes (Apar_B), gDNA from 0B *A. paranae* individuals (Apar_gen), gDNA from *A. fasciatus* specimens (Afasc), with *A. altiparanae* (Aalti) as outgroup.
(TIF)

Figure S2 Maximum likelihood tree built with the H1 gene sequences. The sequences were obtained from the microdissected B chromosomes (Apar_B), gDNA from 0B *A. paranae* individuals (Apar_gen), gDNA from *A. bockmanni* (Abock) and *A. fasciatus* specimens (Afasc), using *A. altiparanae* (Aalti) as outgroup.
(TIF)

Figure S3 Maximum likelihood tree built with the H3 gene sequences. The sequences were obtained from the microdissected B chromosomes (Apar_B), gDNA from 0B *A. paranae* individuals (Apar_gen), gDNA from *A. bockmanni* (Abock) and *A. fasciatus* specimens (Afasc), using *A. altiparanae* (Aalti) as outgroup.
(TIF)

Table S1 DNA sequence of the primers employed for PCR amplification of the different repetitive DNAs assayed.
(DOCX)

Table S2 Codon-based test of neutrality, positive selection and purifying selection for the H1 histone gene partial sequence.
(DOCX)

Table S3 Genetic divergence values among species. Note the higher similarity between the sequences obtained from the B chromosomes (Apar_B) and those from 0B-gDNA in *A. paranae* (Apar_gen) and *A. bockmanni* (Abock), and lower similarity with those from *A. fasciatus* (Afasc) and *A. altiparanae* (Aalti).
(DOCX)
