# Supplementary figures and images for: Delimiting the Origin of a B Chromosome by FISH Mapping, Chromosome Painting and DNA Sequence Analysis in Astyanax paranae (Teleostei, Characiformes)
Source: PLoS One. 2014 Apr 15;9(4):e94896. doi: 10.1371/journal.pone.0094896 (PMC3988084; doi:10.1371/journal.pone.0094896)

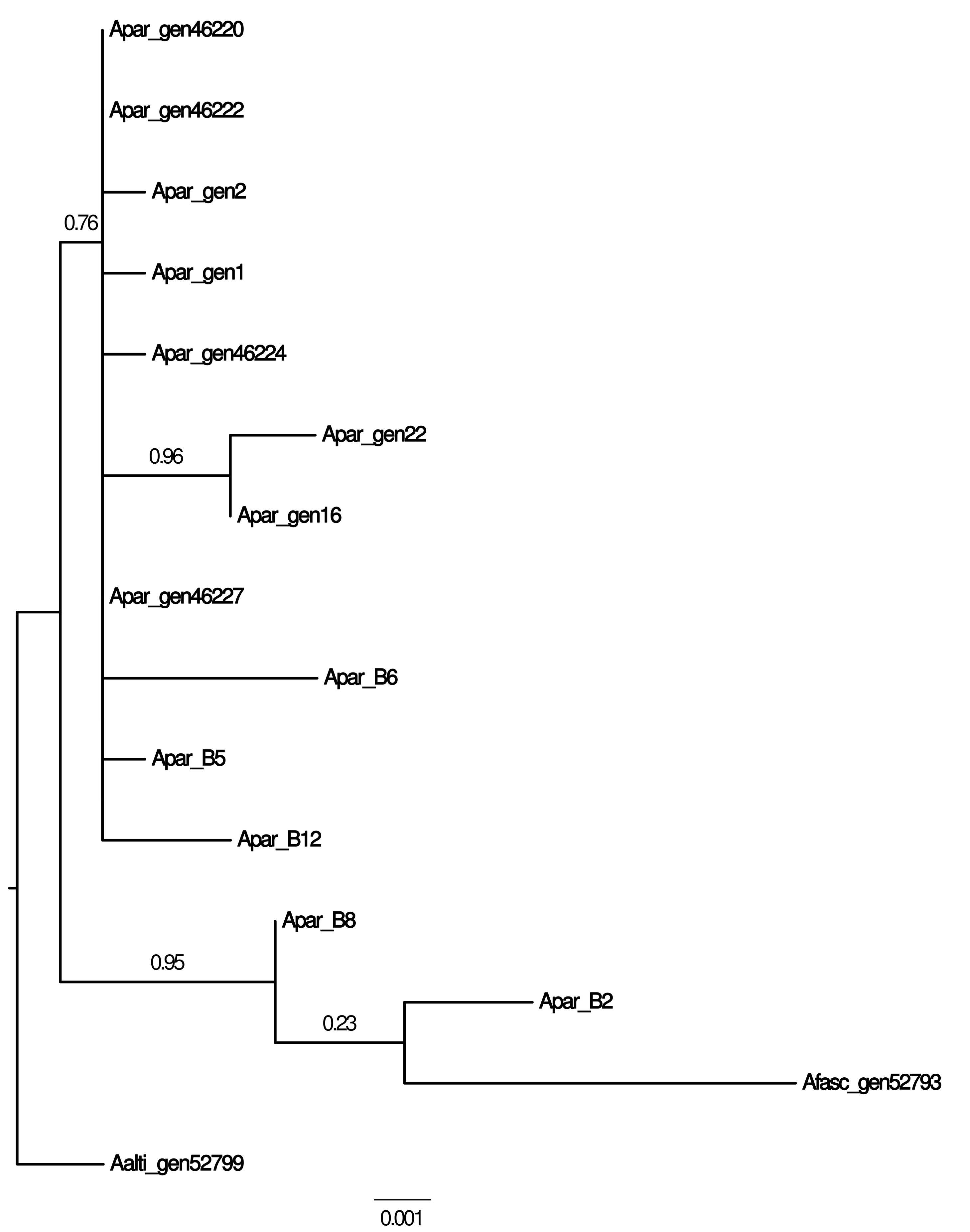

Supplement: Figure S1 — Maximum likelihood tree built with the 18S rDNA sequences. The sequences were obtained from the microdissected B chromosomes (Apar_B), gDNA from 0B A. paranae individuals (Apar_gen), gDNA from A. fasciatus specimens (Afasc), with A. altiparanae (Aalti) as outgroup. (TIF) [file pone.0094896.s001.tif]

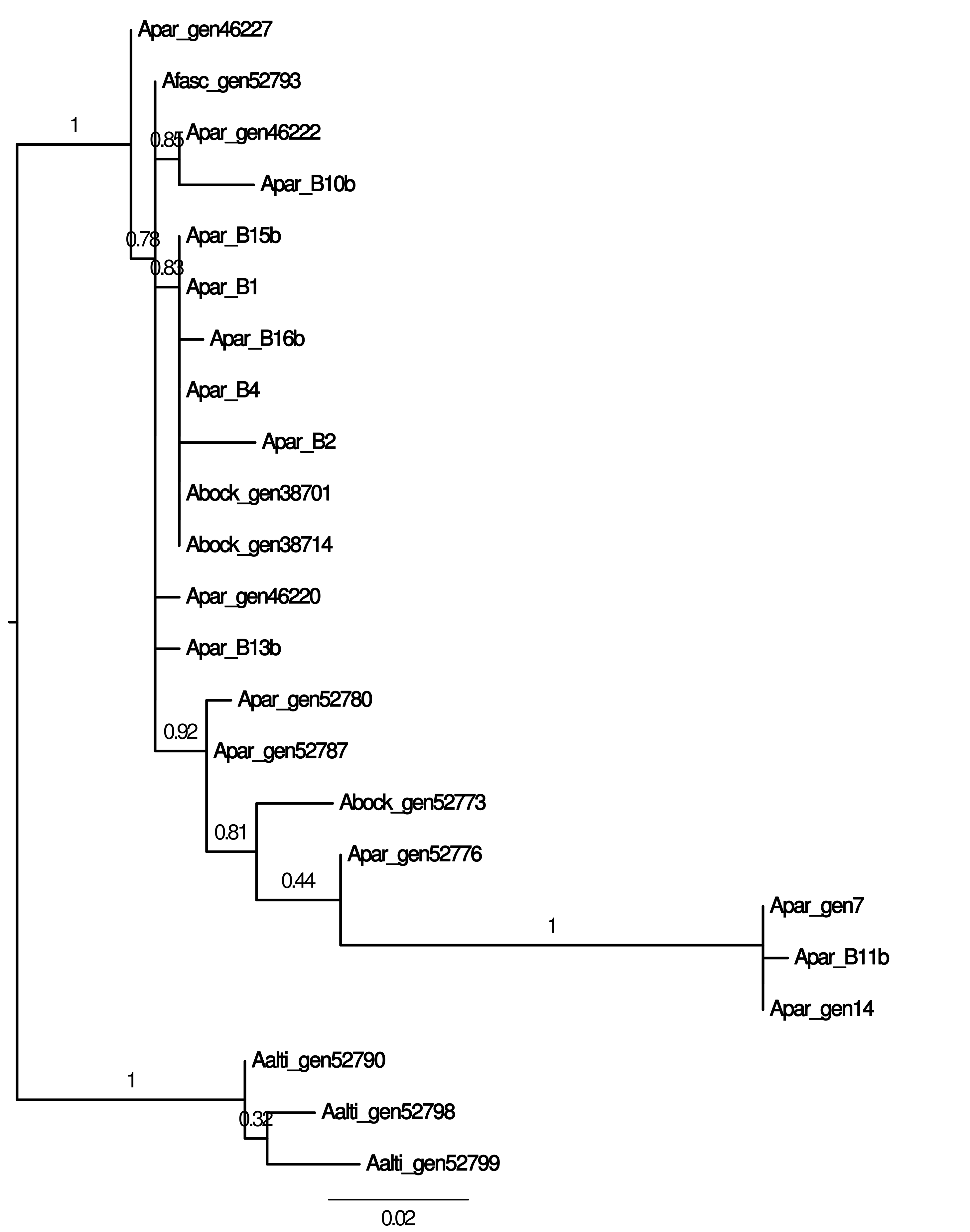

Supplement: Figure S2 — Maximum likelihood tree built with the H1 gene sequences. The sequences were obtained from the microdissected B chromosomes (Apar_B), gDNA from 0B A. paranae individuals (Apar_gen), gDNA from A. bockmanni (Abock) and A. fasciatus specimens (Afasc), using A. altiparanae (Aalti) as outgroup. (TIF) [file pone.0094896.s002.tif]

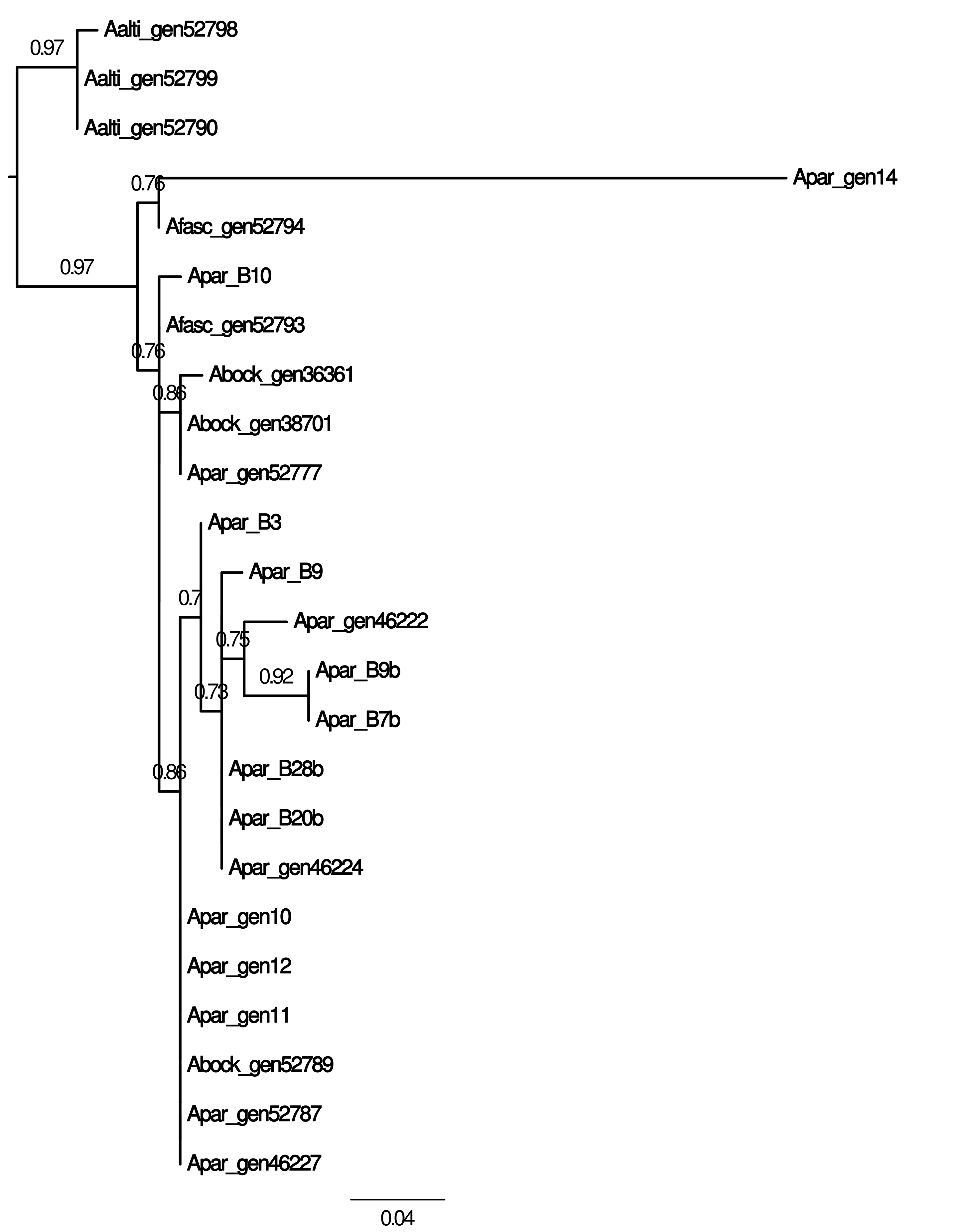

Supplement: Figure S3 — Maximum likelihood tree built with the H3 gene sequences. The sequences were obtained from the microdissected B chromosomes (Apar_B), gDNA from 0B A. paranae individuals (Apar_gen), gDNA from A. bockmanni (Abock) and A. fasciatus specimens (Afasc), using A. altiparanae (Aalti) as outgroup. (TIF) [file pone.0094896.s003.tif]
