# Supplementary material for: Delimiting the Origin of a B Chromosome by FISH Mapping, Chromosome Painting and DNA Sequence Analysis in Astyanax paranae (Teleostei, Characiformes)
Source: PLoS One. 2014 Apr 15;9(4):e94896. doi: 10.1371/journal.pone.0094896 (PMC3988084; doi:10.1371/journal.pone.0094896)
Supplement: Table S1 — DNA sequence of the primers employed for PCR amplification of the different repetitive DNAs assayed. (DOCX) [file pone.0094896.s004.docx]

**Table S1.** DNA sequence of the primers employed for PCR amplification of the different repetitive DNAs assayed.

| Region | Primer  Name | Sequence | Amplicon  Size (bp) | Annealing  Temperature (ºC) | Source |
| --- | --- | --- | --- | --- | --- |
| rDNA 5S | 5S_A | 5’TCAACCAACCACAAAGACATTGGCAC-3’ | 400 | 55 | [1] |
|  | 5S_B | 5’TAGACTTCTGGGTGGCCAAAGGAATCA-3’ |  | 55 |  |
| rDNA 18S | NS1 | 5’GTAGTCATATGCTTGTCTC-3’ | 1839 | 54 | [2] |
|  | NS8 | 5’TCCGCAGGTTCACCTACGGA-3’ |  | 54 |  |
| ITS | SBR | 5′GTAGGTGAACCTGCAGAAGG-3’ | 1670 | 54 | [3] |
|  | JM5 | 5′TACCGGCCTCACACCGTCC-3’ |  | 54 |  |
| H1 histone | H1_F | 5’ATGGCAGAARYCGMCCAG-3’ | 620 | 54 | [4] |
|  | H1_R | 5’TACTTCTTCTTGGGSGCTGC-3’ |  | 54 |  |
| H3 histone | H3_F | 5’ATGGCTCGTACCAAGCAGACVGC-3’ | 374 | 54 | [5] |
|  | H3_R | 5’ATATCCTTRGGCATRATRGTGAC-3’ |  | 54 |  |
| H4 histone | H4F2s | 5′TSCGIGAYAACATYCAGGGIATCAC-3’ | 214 | 58 | [6] |
|  | H4F2er | 5’CKYTTIAGIGCRTAIACCACRTCCAT-3’ |  | 58 |  |
| *Rex*1 retrotransposon | RTX1-F1 | 5’TCCCTCAGCAGAAAGAGTCTGCTC-3’ | 575 | 55 | [7] |
|  | RTX1-R1 | 5’TCCCTCAGCAGAAAGAGTCTGCTC-3’ |  | 55 |  |
| *Rex*3 retrotransposon | RTX3-F1 | 5’CGGTGAYAAAGGGCAGCCCTG-3’ | 325 | 55 | [8] |
|  | RTX3-R1 | 5’TGGCAGACNGGGGTGGTGGT-3’ |  | 55 |  |

1. Pendás AM, Móran P, Freije JP, Garcia-Vásquez E (1994) Chromosomal location and nucleotide sequence of two *tandem* repeats of the Atlantic salmon 5S rDNA. Cytogenetics Cell Genetics. 67: 31–36.
2. White TJ, Bruns T, Lee S, Taylor T (1990) Amplification and direct sequencing of fungal ribosomal RNA for phylogenetics. In: Innis MA, Gelfand DH, Sninsky JJ, White TJ, editors. PCR protocols: a guide to methods and amplications. Academic, San Diego pp 315–322.
3. Montoya-Burgos JI (2003) Historical biogeography of the catfish genus *Hypostomus* (Siluriformes: Loricariidae), with implications on the diversification of Neotropical ichthyofauna. Molecular Ecology 12:1855–1867.
4. Hashimoto DT, Ferguson-Smith MA, Rens W, Foresti F, Porto-Foresti F (2011) Chromosome mapping of H1 histone and 5S RNA gene clusters in three species of *Astyanax* (Teleostei: Characiformes). Cytogenet Genome Res 134: 64–71.
5. Colgan D, McLauchlan A, Wilson G, Livingston S (1998) Histone H3 and U2 snRNA DNA sequences and arthropod molecular evolution. Aust J Zool. 46: 419–43.
6. Pineau P, Henry M, Suspène R et al. (2005)A universal primer set for PCR amplification of nuclear histone H4 genes from all animal species. Mol Biol Evol. 22: 582–588.
7. Volff JN, Körting C, Schartl M (2000) Multiple lineages of the non-LTR retrotransposon *Rex1* with varying success in invading fish genomes. Mol Biol Evol 17: 1673–1684.
8. Volff JN, Körting C, Sweeney K, Schartl M (1999) The non-LTR retrotransposon *Rex3* from the fish *Xiphophorus* is widespread among teleosts. Mol Biol Evol 16: 1427–1438.
