# Supplementary material for: Delimiting the Origin of a B Chromosome by FISH Mapping, Chromosome Painting and DNA Sequence Analysis in Astyanax paranae (Teleostei, Characiformes)
Source: PLoS One. 2014 Apr 15;9(4):e94896. doi: 10.1371/journal.pone.0094896 (PMC3988084; doi:10.1371/journal.pone.0094896)
Supplement: Table S2 — Codon-based test of neutrality, positive selection and purifying selection for the H1 histone gene partial sequence. (DOCX) [file pone.0094896.s005.docx]

**Table S2.** Codon-based test of neutrality, positive selection and purifying selection for the H1 histone gene partial sequence.

| Hypothesis | Z | P |
| --- | --- | --- |
| Neutrality (dN = dS) | -4.391 | <0.001 |
| Positive selection (dN > dS) | -4.184 | 1 |
| Purifying selection (dN < dS) | 4.317 | <0.001 |
