# Supplementary material for: Delimiting the Origin of a B Chromosome by FISH Mapping, Chromosome Painting and DNA Sequence Analysis in Astyanax paranae (Teleostei, Characiformes)
Source: PLoS One. 2014 Apr 15;9(4):e94896. doi: 10.1371/journal.pone.0094896 (PMC3988084; doi:10.1371/journal.pone.0094896)
Supplement: Table S3 — Genetic divergence values among species. Note the higher similarity between the sequences obtained from the B chromosomes (Apar_B) and those from 0B-gDNA in A. paranae (Apar_gen) and A. bockmanni (Abock), and lower similarity with those from A. fasciatus (Afasc) and A. altiparanae (Aalti). (DOCX) [file pone.0094896.s006.docx]

**Table S3.**  Genetic divergence values among species. Note the higher similarity between the sequences obtained from the B chromosomes (Apar_B) and those from 0B-gDNA in *A. paranae* (Apar_gen) and *A. bockmanni* (Abock), and lower similarity with those from *A. fasciatus* (Afasc) and *A. altiparanae* (Aalti).

|  | Apar_gen | Apar_B | Aalti | Abock |
| --- | --- | --- | --- | --- |
| Apar_B | 0.017 |  |  |  |
| Aalti | 0.105 | 0.099 |  |  |
| Abock | 0.035 | 0.032 | 0.109 |  |
| Afasc | 0.048 | 0.046 | 0.113 | 0.051 |
